# Complete Neutralization of Tetanus Neurotoxin by Alpaca-Derived Trivalent Nanobodies Aimed at Veterinary Medical Applications

**DOI:** 10.3390/vetsci13010098

**Published:** 2026-01-19

**Authors:** Chiyomi Sakamoto, Chie Shitada, Norihiko Kiyose, Nobuo Miyazaki, Sena Kamesawa, Hiroshi Morioka, Kazunori Morokuma, Kazuhiko Tomokiyo, Motohide Takahashi

**Affiliations:** 1 Toxin and Biologicals Research Laboratory, Kumamoto Health Science University, 325 Izumi-machi, Kita-ku, Kumamoto 861-5533, Japan; sakamoto.c01@kumamoto-hsu.ac.jp (C.S.); shitada@kumamoto-hsu.ac.jp (C.S.); 2 The Chemo-Sero-Therapeutic Research Institute (KAKETSUKEN), 8-7 Shinshigai, Chuou-ku, Kumamoto 860-0803, Japan; kazuhiko.tomokiyo@kaketsuken.org; 3 ARK Resource Co., Ltd., 383-2 Nakaharamachi, Nishi-ku, Kumamoto 861-5271, Japan; kiyose@ark-resource.co.jp (N.K.); miyazaki@ark-resource.co.jp (N.M.); 4 Graduate School of Pharmaceutical Sciences, Kumamoto University, 5-1 Oe-honmachi, Chuo-ku, Kumamoto 862-0973, Japan; s.kamesawa@seimeibunseki.org (S.K.); morioka@gpo.kumamoto-u.ac.jp (H.M.); 5 KM Biologics Co., Ltd., 1-6-1 Okubo, Kita-ku, Kumamoto 860-8568, Japan; morokuma@kmbiologics.com

**Keywords:** tetanus neurotoxin, VHH, VHH trimer, neutralizing activity, alpaca, neutralization test, One Health

## Abstract

Tetanus is a zoonotic disease posing significant threats to both humans and animals, particularly horses, sheep, and ruminants. Current antitoxin therapies rely on animal-derived immunoglobulins, presenting challenges including animal welfare concerns, pathogen contamination risks, and manufacturing complexity. Alpaca-derived nanobodies (VHH) are promising alternatives owing to their high antigen-binding affinity, thermostability, and potential for microbial production. We developed highly active trivalent VHH antibodies (tVHH) that target multiple epitopes of tetanus neurotoxin (TeNT). Following alpaca immunization with tetanus toxoid, 41 VHH clones were isolated using phage display. Six VHH clones were selected through in vivo neutralization assays, from which three clones of VHH (8, 11, 36) were selected to construct tVHH-8/11/36 and tVHH-8/36/11. Using an improved 21-day mouse neutralization assay, tVHH-8/11/36 demonstrated exceptional neutralizing activity of approximately 1580 IU/mg against 4000 LD_50_ of toxin, substantially exceeding current human and veterinary anti-tetanus immunoglobulin preparations. Surface plasmon resonance and ELISA confirmed that each VHH recognizes different TeNT domains, producing synergistic neutralizing effects through multimerization. Since antitoxin therapy challenges are common to both animals and humans, this tVHH technology supports One Health by providing a unified therapeutic platform applicable across species through sustainable microbial production.

## Simple Summary

Tetanus causes fatal neurological diseases in both humans and animals. In this study, we developed an alpaca-derived trimer, VHH, and achieved high neutralizing activity against tetanus neurotoxin. The tVHH-8/11/36, which links three selected nanobodies, demonstrated a neutralizing activity of approximately 1580 IU/mg, greatly exceeding that of human and veterinary anti-tetanus immunoglobulin preparations. Trimer VHH can be mass-produced in microorganisms, achieving both animal welfare considerations and manufacturing efficiency. This technology is expected to serve as a next-generation antitoxin that embodies the One Health concept, bridging veterinary and human medicine.

## 1. Introduction

Tetanus is a zoonotic disease caused by the neurotoxin produced by *Clostridium tetani*, and represents a significant challenge in veterinary medicine and public health. Bacteria proliferate under anaerobic conditions at wound sites, and the toxin induces severe neurological symptoms. Horses are particularly susceptible to tetanus toxin, with severe cases showing high mortality rates [1,2]. Infection cases have also been reported in ruminants, such as cattle, sheep, and goats, and dogs, through castration, tail docking, and trauma [3,4]. Approximately 34,000 deaths occur annually in humans, primarily in developing countries, necessitating countermeasures from a health perspective [5].

Tetanus neurotoxin (TeNT) is a two-chain toxin with a molecular weight of approximately 150 kDa (1315 amino acids), consisting of a light chain (~50 kDa) with protease activity and a heavy chain (~100 kDa) responsible for binding to nerve cells and membrane translocation connected by a disulfide bond [6,7]. Functionally, the light chain (Fragment A: AA 1-439) acts as a Zn-dependent endopeptidase that cleaves synaptobrevin and inhibits neurotransmission. The heavy chain comprises a membrane translocation domain (Fragment B: AA 440-869) and a neuronal cell membrane-binding domain (Fragment C: AA 870-1315), each presenting distinct neutralizing target epitopes [7].

Currently, equine tetanus antitoxin or human tetanus immunoglobulin (TIG) are primarily used for tetanus treatment. In veterinary medicine, equine tetanus antitoxin is manufactured in Europe, Japan, Australia, and other regions using plasma obtained from horses immunized with tetanus toxoid (TeTd). However, this manufacturing method requires repeated immunization and faces challenges including animal welfare concerns, risk of unknown pathogen contamination, and lengthy production processes [8,9]. In developing countries, where veterinary products are difficult to obtain, human TIG preparations are sometimes used in animals [10]. Human TIG products have been developed as alternatives to equine antitoxin preparations. However, many countries, including Japan, remain dependent on imported manufacturing stock (primarily from the United States), highlighting the need to establish stable domestic supply systems.

In recent years, VHH derived from camelids has attracted attention as a next-generation antibody therapeutic [11,12,13]. Owing to their small size (approximately 1/10 of the molecular weight of conventional IgG (~15 kDa)), VHH are amenable to engineering modifications, possess excellent thermostability and acid resistance, and can be mass-produced in microorganisms (*E. coli*, yeast) [12,13]. These characteristics eliminate the need for lengthy processes such as animal immunization and serum purification, and reduce cold chain requirements, offering significant advantages, particularly for veterinary applications and use in developing countries [13].

Multiple studies have reported on monoclonal antibodies and nanobodies against TeNT; however, when used individually, they exhibit insufficient neutralizing capacity against TeNT. In contrast, combining multiple antibodies that recognize different epitopes has been shown to significantly enhance neutralizing activity [14,15]. However, when multiple antibodies are physically mixed, delayed toxin activity may manifest owing to dissociation in vivo. Conventional TeNT neutralization assays have short observation periods of 3–5 days [16,17], making it difficult to detect such delayed symptoms and accurately evaluate the complete neutralizing capacity of novel antibody formats.

In this study, we selected clones with neutralizing activity from VHH obtained by immunizing alpacas with TeTd, and constructed trivalent VHH (tVHH) genetically linked through protein engineering. A multivalent structure, which simultaneously targets multiple epitopes, was designed to achieve high neutralizing activity and stability as a single molecule. Furthermore, we developed an improved mouse neutralization assay with an extended observation period of 21 days to rigorously evaluate the complete neutralizing capacity of tVHH. If this technology is successfully implemented, it is expected to provide a foundation for next-generation antitoxin therapeutics applicable to both veterinary and human medicine, with consideration for animal welfare, reduced risk of unknown pathogen contamination, and stable manufacturing capabilities.

## 2. Materials and Methods

### 2.1. Alpaca Immunization

Male alpacas (*Vicugna pacos*) were subcutaneously inoculated with an antigen solution consisting of 3 mg/3 mL TeTd (KM Biologics) emulsified with an equal volume of Freund’s complete adjuvant (BD Biosciences, Franklin Lakes, NJ, USA). Subsequently, an antigen solution consisting of 1 mg/mL TeTd emulsified with an equal volume of Freund’s incomplete adjuvant (BD Biosciences, Franklin Lakes, NJ, USA) was subcutaneously administered four times at 14-day intervals. Blood samples (50 mL) collected on days 36 and 64 were treated with an anticoagulant (0.1% EDTA), and peripheral blood mononuclear cells (PBMCs) were isolated by density gradient centrifugation using Leucosep™ lymphocyte separation tubes (Greiner Bio-One, Frickenhausen, Germany). Isolated PBMCs were homogenized using RNAiso Plus (Takara Bio, Shiga, Japan). The collected plasma was separated and purified into IgG1, IgG2, and IgG3 using Protein G and Protein A columns, and antibody titers were confirmed using ELISA.

### 2.2. Detection of Tetanus Antibodies in Immunized Alpaca-Derived Sample by ELISA

High-purity TeTd (500 ng/100 μL per well) was added to 96-well microplates, incubated overnight at 4 °C, and blocked with 0.5% bovine serum albumin (BSA) in PBS. For testing, the plates were washed thrice with PBS containing 0.05% Tween-20 (PBST). Immunized alpaca-derived samples (serum, plasma, IgG1, IgG2, IgG3) were added in stepwise dilutions and incubated at room temperature for 60 min, followed by three washes with the washing buffer. Rabbit anti-alpaca IgG polyclonal antibody diluted in the assay buffer was then added and incubated at room temperature for 60 min. After five washes, HRP-conjugated goat anti-rabbit IgG antibody (Bio-Rad Laboratories, Hercules, CA, USA) was added and incubated at room temperature for 60 min. Following washing, 3,3′,5,5′-tetramethylbenzidine (TMB) substrate solution (Nacalai Tesque, Kyoto, Japan) was added and incubated at room temperature for 10 min. The stop solution was added, and the absorbance at 450/650 nm was measured.

### 2.3. Construction of Immunized Alpaca VHH Phage Library

Total RNA was extracted from the homogenized alpaca PBMCs. cDNA was synthesized from 5 μg total RNA by reverse transcription using SuperScript III (Thermo Fisher Scientific, Waltham, MA, USA). First-round PCR was performed using KOD-Plus DNA polymerase (Toyobo, Osaka, Japan), with 5′ primers 1st-F1 (5′-AGKTGCAGCTCGTGGAGTCNGGNGG-3′) and 1st-F2 (5′-AGGTGCAGCTCGTGGAGTCTGGGGG-3′) and the 3′ primer 1st-R (5′-TTGTGGTTTTGGTGTCTTGGG-3′). Second-round PCR, to add the SfiI and SpeI restriction enzyme sites to the 5′- and 3′-termini of the first PCR product, respectively, was performed using GeneTaq DNA polymerase (Nippon Gene, Tokyo, Japan), with 5′ primer 2nd-F (5′-TGCTCCTCGCGGCCCAGCCGGCCATGGCTCAGGTGCAGCTCGTGGAGTCTGGGGG-3′) and the 3′ primer 2nd-R (5′-ATGATGATGTGCACTAGTTTGTGGTTTTGGTGTCTTGGG-3′).

The second-round PCR products and the phagemid vector, both purified by agarose gel electrophoresis, were digested with restriction enzymes, and the PCR products were ligated into a linearized phagemid vector (pKSTV03). The ligated plasmids were purified by phenol/chloroform extraction and ethanol precipitation and then transformed into *Escherichia coli* (*E. coli*) TG-1 cells (Lucigen, Middleton, WI, USA) by electroporation. For phage construction, transformed *E. coli* TG-1 cells were cultured in 2TYAG medium (containing 16 g tryptone, 10 g yeast extract, 5 g NaCl, 2% glucose, and 100 mg ampicillin sodium in a final volume of 1 L) and infected with M13KO7 helper phage. The flask was shaken for 30 min before adding kanamycin (50 mg/L). The culture supernatant containing the constructed phage was collected by centrifugation, and 0.2 volumes of 20% PEG/NaCl (20% polyethylene glycol 6000 solution containing 2.5 M NaCl) was added. The phage was precipitated at 4 °C for 6 h, and the pellet was collected by centrifugation. The pellet was resuspended in PBS, and the supernatant obtained after centrifugation was used for biopanning or other assays.

### 2.4. Selection of Antigen-Specific VHH by Biopanning

Enrichment of antigen-specific phage populations was performed by biopanning. TeNT was provided by KM Biologics Co., Ltd. Tetanus toxin fragments were prepared as follows: for fragments A and BC, purified TeNT was incubated at 37 °C for 2–5 h with 1 M DTT, then mixed with an equal volume of 4 M urea (FUJIFILM Wako, Osaka, Japan). This mixture was gel-filtered through two HiPrep™ 16/60 Se-phacryl™ S-200 columns (Cytiva, Tokyo, Japan) connected in series. The first peak was designated as fragment BC (heavy chain), and the second peak as fragment A (light chain). For fragment C preparation, 3 mg of purified TeNT was incubated at 37 °C for 18 h in a buffer containing 5 μg/mL papain (MP Bio, Tokyo, Japan), 1 mM EDTA (FUJIFILM Wako, Osaka, Japan), 0.9 mM cysteine (FUJIFILM Wako, Osaka, Japan), and 20 mM phosphate buffer (pH 6.5). A 1000-fold molar excess of antipain (Sig-ma-ALDRICH, MO, USA) was added to inhibit papain activity, and the mixture was gel-filtered using HiLoad 16/60 Superdx 200 pg (Cytiva, Tokyo, Japan). The third peak among the three peaks was designated as fragment C.

For biopanning, immunoplates were coated with 1–5 μg/150 μL of antigen (TeNT, Fragment A, Fragment BC, or Fragment C) overnight at 4 °C, followed by blocking with a blocking reagent. After washing thrice with PBST (0.05% Tween-20 in PBS), the phage library was added and incubated for 90 min. After five washes with PBST, antigen-bound phages were eluted with 0.1 M glycine-HCl (pH 2.2). The eluate was neutralized by adding 1 M Tris-HCl (pH 9.1) and used to infect *E. coli* TG-1 cells. Infected *E. coli* TG-1 cells were cultured to construct a library. Phage construction followed the same procedure as described above. Two rounds of biopanning were conducted using the constructed phage library. After biopanning, the library was plated on 2TYAG plates and single colonies were cultured. Phages were constructed and clones were isolated following the phage preparation procedure described above.

### 2.5. Expression and Purification of VHH

Plasmids from the phage clones were transformed into *E. coli* HB2151 cells by heat shock and grown on 2TYAG plates. Single colonies were cultured in 2TYA medium and 1 mM isopropyl β-D-thiogalactopyranoside (IPTG) (Takara Bio, Shiga, Japan) was added, followed by overnight culture at 37 °C. The culture supernatant was collected by centrifugation, and the pellet was resuspended in TES buffer (0.2 M Tris, 0.5 mM EDTA, 0.5 M sucrose). After adding 1/4 volume of TES buffer, the periplasmic fraction was recovered. Both the culture supernatant and periplasmic fraction samples were applied to a Ni column (Cytiva, Marlborough, MA, USA), and the VHH antibodies were purified using the C-terminal His-tag fused to the VHH. The purified VHH were dialyzed against PBS and used for various assays.

### 2.6. Evaluation of Antigen-Binding Activity of Phage and VHH by ELISA

The 96-well plates were coated with 250 ng/50 μL per well of antigen per well and incubated overnight at 4 °C, followed by blocking with 0.5% BSA in PBS). After washing thrice with PBST, phage or VHH solution was added and incubated for 60 min. Following three washes with PBST, biotinylated anti-M13 phage mouse antibody (Abcam, Cambridge, UK) and HRP-conjugated streptavidin (Vector Laboratories, Burlingame, CA, USA) were used for phage detection. Biotinylated anti-His-tag mouse antibody (MBL, Nagoya, Japan) and HRP-conjugated anti-mouse antibody (KPL, Gaithersburg, MD, USA) were used for VHH detection. TMB was used as the substrate.

### 2.7. Design and Construction of VHH Trimers

Two clones of tVHH were constructed using three clones of VHH (8, 11, 36) that exhibited neutralizing activity: tVHH-8/11/36 (arranged in the order VHH-8, VHH-11, and VHH-36 from the N-terminus to the C-terminus) and tVHH-8/36/11 (arranged in the order VHH-8, VHH-36, and VHH-11 from the N-terminus to the C-terminus). The VHH trimers were designed to be linked using a flexible GS linker [(GGGGS)_3_] composed of repeating glycine and serine residues to avoid affecting the three-dimensional structure of each connected VHH [18]. The designed genes were artificially synthesized. VHH trimers were ligated into the vector pET22b (+) vector using the restriction enzymes SfiI and SpeI. The constructed plasmids were transformed into *E. coli* BL21 cells by heat shock and grown on 2TYAG agar. Subsequent procedures were the same as described in “2.5 Expression and Purification of VHH.”

### 2.8. Expression and Purification of GST-VHH

Genes encoding the GST-VHH fusion proteins were synthetically designed and cloned into the pGEX-5X-1 vector. The constructed plasmids were transformed into *E. coli* BL21 cells using heat shock and grown on 2TYAG agar plates. Single colonies were cultured in 2TYA medium and protein expression was induced by the addition of 1 mM IPTG followed by incubation at 37 °C for 4 h. After centrifugation, the cell pellet was resuspended in suspension buffer (20 mM Tris-HCl, 500 mM NaCl, and 1 mM DTT, pH 7.5) and disrupted by sonication. The supernatant obtained after centrifugation was applied to a glutathione Sepharose column (Cytiva, Marlborough, MA, USA) and GST-VHH fusion proteins (Figure S1) were purified using an N-terminal GST tag. Purified proteins were dialyzed against PBS and used for subsequent assays.

### 2.9. Surface Plasmon Resonance (SPR) Analysis

The binding affinity between VHH and TeNT and its fragments was evaluated using the Biacore T200 system (Cytiva, Marlborough, MA, USA) at 25 °C. Anti-glutathione S-transferase (GST) antibody was immobilized on a CM5 sensor chip (Cytiva, Marlborough, MA, USA) as a capture molecule, followed by reaction with three clones of VHH (8, 11, 36) fused with GST tags as ligands. Subsequently, binding with purified TeNT and fragments A and C as analytes was confirmed. Each measurement was performed in triplicate using HBS-EP running buffer (10mM HEPES pH 7.4, 150mM NaCl, 3mM EDTA, and 0.005% Tween 20). The dissociation constant (*K*_D_ value) was calculated by fitting to a single-site binding model using the Biacore T200 Evaluation Software 3.0 (Cytiva, Marlborough, MA, USA).

### 2.10. Analysis of Binding Activity by ELISA

ELISA was performed to evaluate the binding specificity of VHH monomers (VHH-8, VHH-11, and VHH-36) to various antigen fragments. VHH adjusted to 10 ng/mL (100 μL per well) and antigen solution (TeTd, TeNT, H chain, L chain, Fragment C) adjusted to 50 ng/mL (100 μL per well) were added to plates coated with rabbit anti-tetanus polyclonal antibody and incubated at 37 °C for 120 min. After washing the plates, peroxidase AffiniPure Goat Anti-Alpaca IgG, VHH domain (Fujifilm Wako, Osaka, Japan) was added and incubated at 37 °C for 60 min. Following plate washing, substrate solution was added and incubated at 37 °C for 30 min. The reaction was stopped with a stop solution and the absorbance was measured at 450/630 nm. The antigen fragments used were fractionated using TeNT.

### 2.11. Mouse Neutralization Test

TeNT neutralizing antibody titers were measured using a mouse neutralization test based on Japanese Minimum Requirements for Biological Products. The national standard for tetanus test toxin used in the test was purchased from the National Institute of Infectious Diseases (NIID, lot no. 5). Toxin doses were expressed as median lethal dose (LD_50_), the dose required to kill 50% of test animals. The L+ dose (Limes Tod, or limit of death) represents the smallest amount of toxin that, when mixed with one standard unit of antitoxin, kills a test animal. The test doses were expressed as fractions of the L+ dose: L+/10 represents one-tenth of the L+ dose (4000 LD_50_ per mouse), L+/100 represents one-hundredth (400 LD_50_), L+/1000 represents one-thousandth (40 LD_50_), and L+/10,000 represents one ten-thousandth (4 LD_50_).

According to this standard, test samples or national standard for tetanus antitoxin (NIID, lot no. 5) were serially diluted in five steps using 0.2% gelatin-PBS, centered at 0.1 units. These diluted samples were mixed with L+/10 level toxin (4000 LD_50_ per mouse). The toxin-antibody mixture (0.4 mL) was subcutaneously injected into the left medial thigh of mice aged 23–29 days. Mice were evaluated in groups of four. However, owing to the limited quantity of antibody, preliminary neutralizing titer evaluations of VHH monomers were conducted using lower toxin doses (L+/100, L+/1000, L+/10,000; two mice per group), with toxin amounts adjusted stepwise starting from L+/10,000 (4 LD_50_).

To evaluate the tVHH trimers, an improved mouse neutralization test with an extended observation period of 21 days was performed. Toxin at the L+/10 level (4000 LD_50_/mouse) was mixed with serially diluted tVHH or standard antitoxin in 0.2% gelatin-PBS and evaluated in groups of four mice. Mice were administered 0.4 mL of the toxin-tVHH mixture, and onset and mortality were observed for 21 days. The severity and duration of onset were scored using a seven-level scoring system (0 = death to 5 = asymptomatic) to quantitatively evaluate the neutralizing activity (Table 1). The neutralizing activity of tVHH was calculated as the relative potency against the standard antitoxin using the probit method in Statistical Analysis software Version 11 (NIID).

This study was approved by the Animal Experiment Committee of Kumamoto Health Science University (approval number: 20-02, 23-09) and was conducted in accordance with animal welfare guidelines.

## 3. Results

### 3.1. Alpaca Immunization and Antibody Response

**Figure 1:**
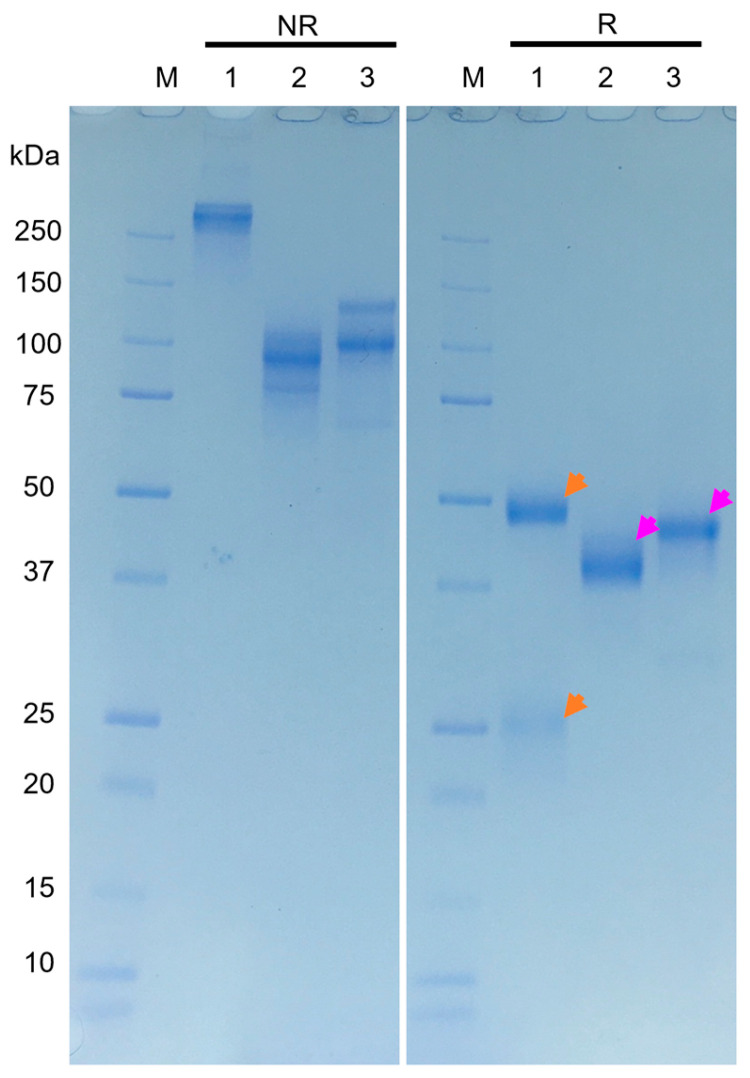
SDS-PAGE Analysis of Purified Antibodies under Reducing and Non-Reducing Conditions. IgG1, IgG2, and IgG3 purified from immunized alpaca plasma using Protein G and Protein A columns were separated on a 5–20% gradient polyacrylamide gel and stained with Coomassie Brilliant Blue. Lane M: molecular weight marker (10–250 kDa); Lane 1: IgG1; Lane 2: IgG2; Lane 3: IgG3. Samples were analyzed under non-reducing conditions (NR) or reducing conditions with 10 mM dithiothreitol at 100 °C for 5 min (R). Under non-reducing conditions, IgG1 (lane 1) showed a high–molecular-weight band (>250 kDa), which resolved into heavy (~50 kDa) and light (~25 kDa) chains under reducing conditions (orange arrows), indicating covalent aggregates. IgG2 (lane 2) appeared as a ~100 kDa band and as a single heavy chain after reduction (pink arrows), consistent with a heavy-chain-only structure. IgG3 (lane 3) showed two bands (~100 and 120 kDa) under non-reducing conditions that converged into a single heavy chain upon reduction (pink arrows), reflecting structural heterogeneity rather than aggregation. IgG3 migrated at a higher apparent molecular weight than IgG2, consistent with its longer hinge region. Each lane contained 1 μg of protein.

Following five immunizations of alpacas with TeTd, anti-tetanus antibody titers in plasma increased as measured by ELISA. The plasma collected on day 64 showed significantly elevated anti-tetanus antibody levels compared to pre-immunization samples. The collected plasma was separated and purified into IgG1, IgG2, and IgG3 using Protein G and Protein A columns, and the fractionation was confirmed by SDS-PAGE (Figure 1 and Figure S2). A marked increase in IgG1 antibody levels and a particularly pronounced elevation in the heavy-chain antibody IgG3 fraction were observed. In contrast, the heavy-chain antibody IgG2 fraction showed minimal elevation.

### 3.2. VHH Selection from Phage Display Library

The size of the phage display library constructed from PBMCs collected on day 64 was 8.23 × 10^8^ colony-forming units. After two rounds of biopanning using four types of antigens (TeNT, Fragment A, Fragment BC, and Fragment C), 41 VHH clones that specifically bound to TeTd were obtained. Amino acid sequence analysis of these clones confirmed sequence diversity, with mutations concentrated in the complementarity-determining regions (CDRs).

### 3.3. Screening by In Vivo Neutralization Test

**Figure 2:**
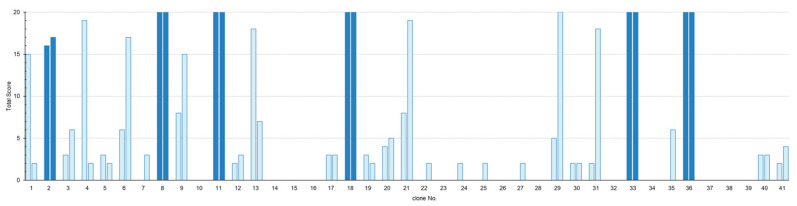
Neutralization Assay Screening of VHH Clones. Forty-one VHHs clones were screened for neutralizing activity against L+/10,000 (4 LD_50_) of TeNT. Each clone was tested in two mice, with symptoms monitored on days 4, 7, 10, and 14 post-inoculation, and scored from zero (death/severe) to five (asymptomatic) per observation day. Bars represent the total scores for both mice across all four time points (maximum, 20 points). 14 VHH clones demonstrated neutralizing activity with scores ≥10. Among these, six VHH clones indicated by the dark-colored bars resulted in survival in both mice. Five of these VHH clones (VHH-8, -11, -18, -33, and -36) demonstrated complete protective efficacy (score ≥20). VHH-2 resulted in survival despite extremely mild disease.

Forty-one monomeric VHH clones were expressed in *E. coli*, purified, and evaluated in preliminary neutralization tests at the L₊/10,000 level (4 LD_50_). Based on the sum of scores on days, 4, 7, 10, and 14 of observation, six VHH clones demonstrated neutralizing activity (Figure 2). From these six VHH clones showing neutralizing activity, neutralization was further evaluated by progressively increasing toxin levels to L₊/1000 (40LD_50_) and L₊/100 (400 LD_50_), resulting in the selection of three clones of VHH (8, 11, 36).

### 3.4. Amino Acid Sequence Analysis of the Three Selected VHH Clones

**Figure 3:**
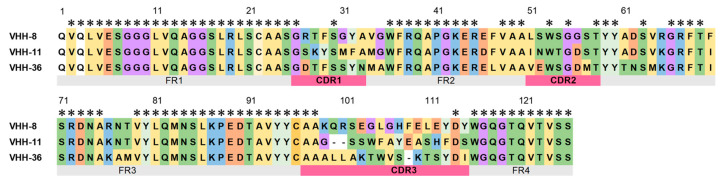
Amino Acid Sequence Alignment of Selected VHHs. The framework regions (FR1, FR2, FR3, and FR4) are shown in gray, and the complementarity-determining regions (CDR1, CDR2, and CDR3) are shown in red. VHH-8 and VHH-11 showed partial sequence similarity in the CDR2 region, whereas VHH-36 exhibited a markedly different sequence pattern across all CDR regions. Particularly in the CDR3 region, the chain length and sequence differed among three VHH clones. Asterisks (*) indicate perfectly matched residues. Coloring indicates physicochemical similarity of residues according to the default MEGA11 (Molecular Evolutionary Genetics Analysis Version 11) coloring scheme.

Amino acid sequence analysis of the three clones of VHH (8, 11, 36) revealed that VHH-8 and VHH-11 showed partial sequence similarity in the CDR2 region, but their sequences in the CDR1 and CDR3 regions were clearly distinct (Figure 3). VHH-36 exhibited a sequence pattern markedly different from those of VHH-8 and VHH-11, with notable sequence diversity observed across all regions from CDR1 to CDR3. In all three VHH clones, amino acid substitutions characteristic of camelid VHH were conserved in the framework regions.

### 3.5. SPR Analysis

**Figure 4:**
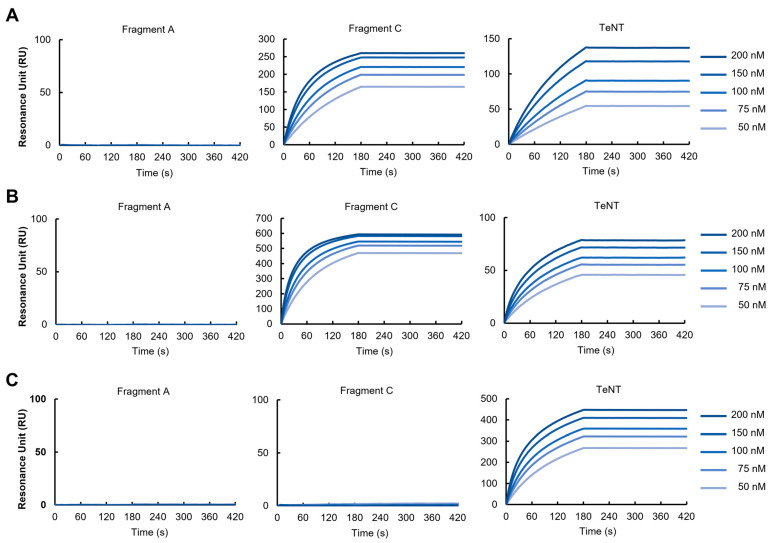
SPR Analysis of VHH Binding to Tetanus Neurotoxin. Anti-GST antibodies were immobilized on a CM5 sensor chip, and GST-VHHs (8, 11, 36) was used as a ligand at 200 RU. Purified TeNT, Fragment A, and Fragment C were injected as the analytes at a flow rate of 50 μL/min for 180 s. HBS-EP was used as the running buffer. Binding sensorgrams for Fragment A (left panels), Fragment C (middle panels), and full-length TeNT (right panels) at analyte concentrations of 50, 75, 100, 150, and 200 nM are shown. The *x*-axis represents time (s) and the *y*-axis represents the resonance units (RU). (**A**) VHH-8 exhibited high-affinity binding (*K_D_* < 10^−11^ M) to both full-length TeNT and Fragment C, but did not bind to Fragment A. (**B**) VHH-11 exhibited high-affinity binding (*K_D_* < 10^−11^ M) to full-length TeNT and Fragment C, but did not bind to Fragment A. (**C**) VHH-36 exhibited high-affinity binding (*K_D_* < 10^−11^ M) to full-length TeNT, but did not bind to Fragment A or Fragment C.

The binding affinity of the three clones of VHH (8, 11, and 36) to TeNT and its fragments was quantitatively evaluated by SPR. Both VHH-11 and VHH-8 demonstrated affinity with *K*_D_ < 10^−11^ M for both full-length TeNT and Fragment C (Figure 4). VHH-11 showed a slightly higher affinity than VHH-8. VHH did not exhibit detectable binding to Fragment A (light chain). VHH-36 bound to full-length TeNT with *K*_D_ < 10^−11^ M, but no significant binding to Fragment C or Fragment A observed. All three VHH exhibited extremely high affinities (*K*_D_ < 10^−11^ M) for full-length TeNT.

### 3.6. Confirmation of Binding Specificity by ELISA

VHH-8 showed the strongest binding to full-length TeNT, followed by Fragment C alone and Fragment BC. Binding to Fragment A occurred at the background level. VHH-11 demonstrated equally strong binding to Fragment C, Fragment BC, and full-length TeNT. VHH-11 also showed minimal binding to Fragment A. VHH-36 exhibited the strongest binding affinity for full-length TeNT, but showed weak binding to Fragment BC and minimal binding to Fragment C alone or Fragment A alone.

### 3.7. Neutralizing Activity of Monomers and Mixtures

**Figure 5:**
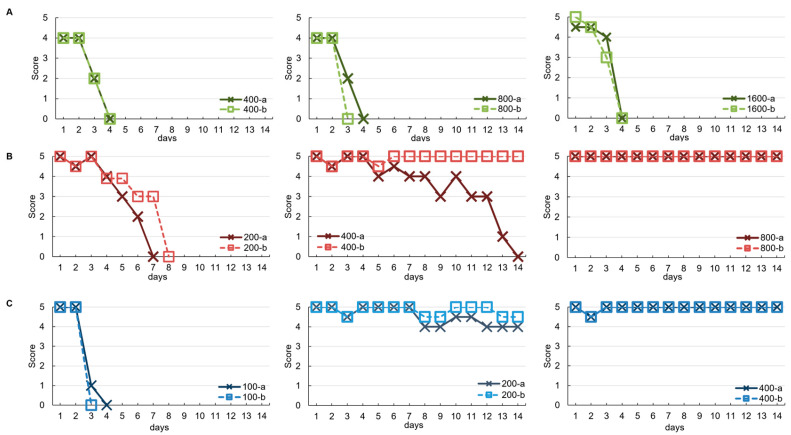
In Vivo Neutralization Test of VHH Monomers. Mice (two per dose, designated as mouse a [×] and mouse b [□]) were administered 40 LD_50_ of TeNT mixed with VHH and observed for 14 days. Symptom score: 0 = death, 1 = severe paralysis, 2–4 = progressive symptoms, 5 = normal. (**A**) VHH-8 (green): Testing at 400, 800, and 1600 μg/mouse resulted in rapid symptom onset at all doses, with death occurring within 2–3 days. (**B**) VHH-11 (red): At 200 μg/mouse, death occurred on days 7 and 8. At 400 μg/mouse, one mouse developed symptoms after day 7 and died on day 14. At 800 μg/mouse, no symptoms were observed and both mice survived. (**C**) VHH-36 (blue): At 200 and 400 μg/mouse, complete protective efficacy was demonstrated throughout the observation period, confirming that VHH-36 possessed the most potent neutralizing activity among the three VHH monomers.

**Figure 6:**
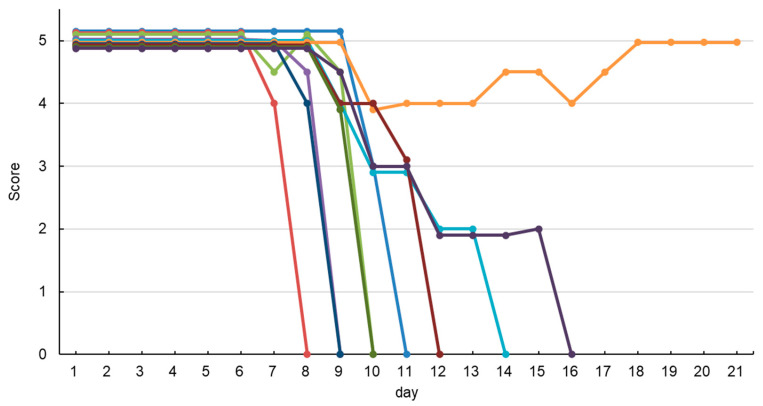
In Vivo Neutralization Test of VHH Monomer Mixture. VHH-8, VHH-11, and VHH-36 monomers were mixed in equal amounts (0.3 mg each, total 0.9 mg) and reacted with TeNT at L+/100 (400 LD_50_). This reaction mixture (0.4 mL containing 0.9 mg VHH and 400 LD_50_ TeNT per mouse) was subcutaneously injected into ten mice, which were observed for 21 days. Each line represents an individual mouse. Symptom scores (0 = death, 5 = normal) were recorded daily. No symptoms were observed until day 6. Symptoms began appearing on day 7, and by day 16, nine out of ten mice had died. Only one mouse (orange line) survived the entire 21-day observation period.

In the L+/1000 (40 LD_50_) test, VHH-11 neutralized the toxin at 400 μg, corresponding to an estimated 0.0025 U/mg, whereas VHH-36 neutralized at 200 μg, corresponding to an estimated 0.005 U/mg. VHH-8 showed no neutralizing effect even at 1600 μg, suggesting an activity of less than 0.0006 U/mg (Figure 5, Table 2). In mice administered with VHH, atypical symptom patterns such as generalized paralysis and muscle contractions were observed, rather than the localized hind limb spastic paralysis characteristic of typical tetanus symptoms. In evaluations combining multiple VHH, when VHH-36 at 30 μg, VHH-11 at 15 μg, and VHH-8 at 15 μg were mixed in this ratio (total 60 μg), the mixture was able to neutralize 40 LD_50_ of toxin at the L+/1000 level (40 LD_50_). At higher toxin levels (L+/100 level, 400 LD_50_), even when three VHH clones were mixed at 300 μg each (total 900 μg), symptoms appeared in all mice, and ultimately nine out of 10 mice died. When a total of 0.9 mg of VHH-8, VHH-11, and VHH-36 were used, the neutralizing antibody titer at the L+/100 level was estimated to be less than 0.01 U/mg. The atypical symptoms were completely undetected by conventional field observation and became prominent after day 7 (Figure 6).

### 3.8. Design and Production of tVHH

**Figure 7:**
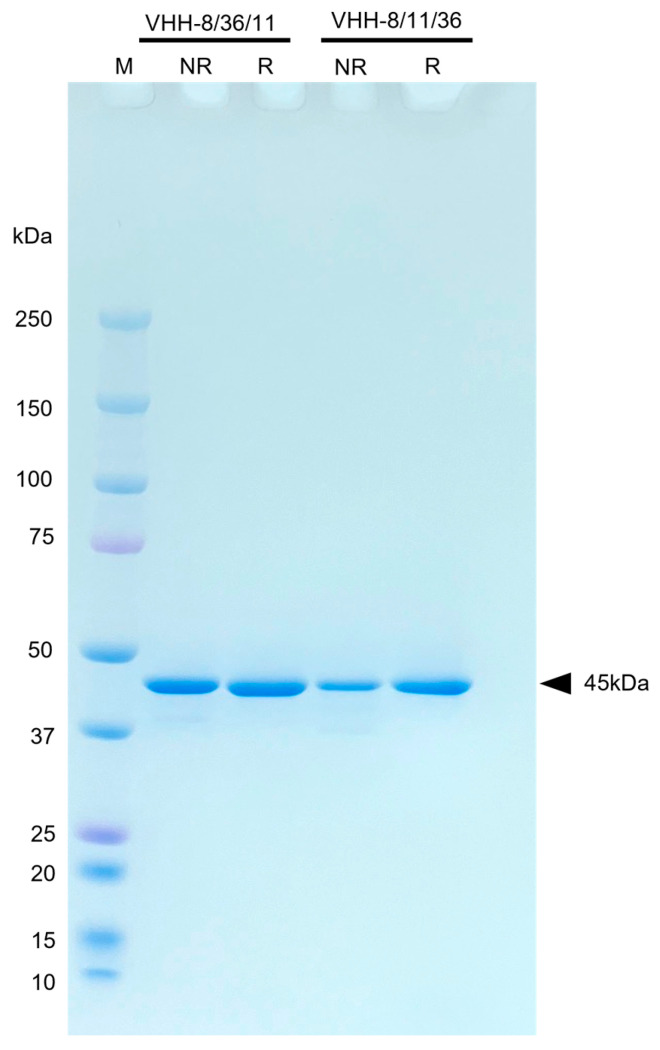
SDS-PAGE Analysis of tVHH under Reducing and Non-Reducing Conditions. tVHH-8/36/11 and tVHH-8/11/36 purified from the supernatant and periplasmic fraction of *E. coli* BL21 were separated on a 5–20% Gradient polyacrylamide gel and stained with Coomassie Brilliant Blue. Lane M: molecular weight marker (10–250 kDa); Samples were analyzed under non-reducing conditions (NR) or reducing conditions with 10 mM DTT at 100 °C for 5 min (R). Both VHH trimers migrated as single bands at approximately 45 kDa (indicated by arrow, expected molecular weight: ~45 kDa), indicating high purity. 3 μg of protein was loaded per lane.

SDS-PAGE analysis confirmed a major band at the expected molecular weight of approximately 45 kDa (Figure 7 and Figure S3). Expression yields were approximately 5 mg/L culture for tVHH-8/11/36 and approximately 4 mg/L culture for tVHH-8/36/11.

### 3.9. Binding Properties of tVHH

**Figure 8:**
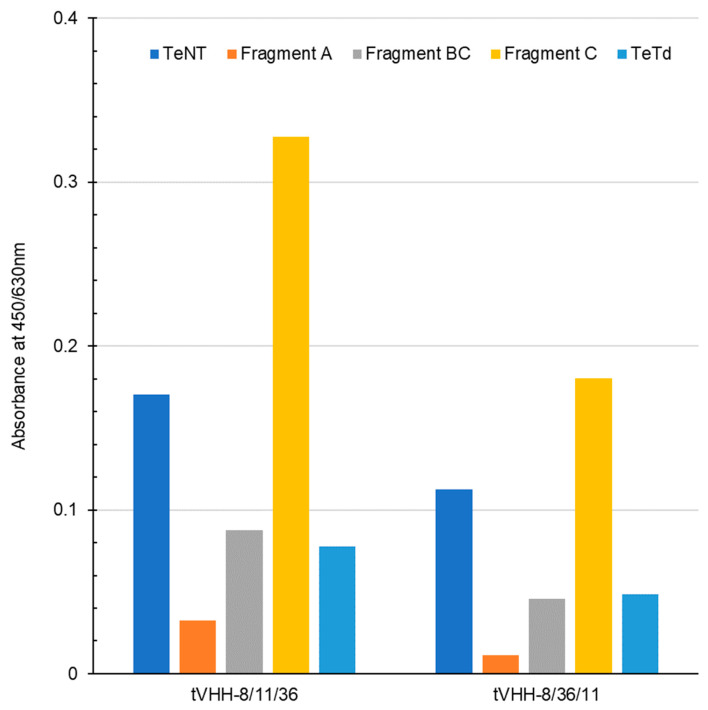
ELISA Binding Analysis of tVHH to Tetanus Neurotoxin and Fragments. Binding of tVHH-8/11/36 and tVHH-8/36/11 to full-length TeNT (blue), toxin fragments (Fragment A, orange; Fragment BC, gray; Fragment C, yellow), and TeTd (light blue) was assessed by ELISA. Absorbance at 450/630 nm indicates binding activity. Both tVHH trimers showed strongest binding to Fragment C, with tVHH-8/11/36 exhibiting higher binding activity than tVHH-8/36/11. Moderate binding to full-length TeNT was observed, while minimal binding was detected to Fragment A, Fragment BC, and TeTd.

The binding activity of tVHH to various antigens was evaluated by ELISA (Figure 8). tVHH-8/11/36 showed strong binding to TeNT, Fragment C, and Fragment BC, confirming that the binding characteristics of the constituent monomers were retained.

### 3.10. Neutralizing Activity of tVHH

Results from the improved mouse neutralization test showed that tVHH-8/11/36 exhibited a neutralizing activity of approximately 1583 units per mg of protein. In contrast, the estimated relative potency of tVHH-8/36/11 was approximately 1056 units/mg. A slight difference in neutralizing activity was observed between the two trimers owing to differences in the linkage order. During the 21-day observation period, no onset or mortality was observed with either tVHH after day 8.

## 4. Discussion

We developed trivalent VHH antibodies targeting multiple epitopes of tetanus neurotoxin and achieved exceptionally high neutralizing activity. The tVHH-8/11/36 demonstrated a neutralizing activity of approximately 1580 IU/mg against 4000 LD_50_ of toxin, substantially exceeding current human and veterinary anti-tetanus immunoglobulin preparations. This represents a significant advance, as single monoclonal antibodies typically exhibit insufficient neutralizing capacity against TeNT [14,15].

The high neutralizing activity of our tVHH can be attributed to its multi-epitope targeting strategy, which addresses the complex structure of TeNT. TeNT is a multi-domain toxin requiring neutralization of multiple functional steps including receptor binding (Fragment C), membrane translocation (Fragment B), and protease activity (Fragment A) [6,7].

SPR analysis revealed that VHH-8 and VHH-11 exhibit extremely high affinity for Fragment C, with K_D values below 10^−11^ M. In contrast, VHH-36 showed similarly high affinity (K_D < 10^−11^ M) for the full-length TeNT, but no detectable binding was observed to the isolated Fragment A or Fragment C. In the ELISA, VHH-36 showed very weak binding to both Fragment B and Fragment C, but did not bind to Fragment A. On the other hand, it exhibited very strong binding activity to the toxin. These findings suggest that VHH-36 recognizes a conformational epitope formed by Fragments B and C together, rather than recognizing them individually.

The two clones of tVHH produced in this study consist of the same VHH but differ in linkage order, with differences observed in neutralizing activity. In tVHH-8/11/36, which showed higher activity, the consecutive arrangement of two Fragment C-binding VHH (VHH-8 and VHH-11) is thought to promote cooperative binding to Fragment C, enabling more robust toxin capture. This arrangement also allows VHH-36, positioned at the C-terminus, to efficiently access its conformational epitope on the full-length TeNT. In contrast, in tVHH-8/36/11, VHH-36 is positioned centrally, physically separating the two Fragment C-binding VHH, potentially slightly reducing the cooperative binding effect.

Previous studies have consistently demonstrated the importance of multi-epitope targeting for TeNT neutralization. Yousefi et al. reported that monoclonal antibodies binding to Fragment C are critical for toxin neutralization and inhibition of TeNT binding to GT1b ganglioside [14]. Aliprandini et al. showed that combining three human monoclonal antibodies enabled complete neutralization at doses where no effect was observed individually [15]. Yasui et al. identified Fragment B-affinity VHH as well as Fragment C involved in GT1b binding inhibition and mouse neutralization capacity [19]. Hans et al. demonstrated that VHH multimerization improves neutralizing activity against TeNT, with multimers containing two different TeNT-binding VHH showing more than 10-fold improved affinity compared to single-VHH multimers [20]. These findings support our multi-epitope targeting strategy and demonstrate that combining antibodies against different functional domains significantly enhances neutralizing activity.

A critical finding of this study was the importance of extended observation periods in neutralization assays. Conventional tetanus antitoxin evaluation methods use short observation periods: in Japan, a five-day evaluation uses lethality as an indicator [17], while the European Pharmacopoeia uses a four-day evaluation with paralysis as an indicator [16]. However, in our mouse neutralization tests of VHH monomers and dimers, we observed paralysis and death occurring after day 7, which would not be detected by these standard protocols. This delayed toxicity is presumed to result from gradual dissociation of toxin-antibody complexes in vivo, with released toxin exhibiting delayed activity.

Notably, our extended observation revealed a critical distinction between physically mixed VHH combinations and genetically linked tVHH. While physically mixed combinations initially appeared protective, symptoms emerged after day 7 with 90% mortality by day 16, indicating progressive dissociation of the antibody mixture from the toxin. In stark contrast, the genetically linked tVHH-8/11/36 provided complete and sustained protection with no symptoms observed after day 8. This demonstrates that covalent linkage maintains the multi-epitope binding configuration necessary for complete neutralization, preventing the gradual dissociation observed with physical mixtures.

Past studies using monoclonal antibodies against TeNT have employed varying observation periods—Yousefi et al. used 14 days [14], Hans et al. 96 h [20], Wang et al. seven days [21,22], and Minamitani et al. 48 h [19]—but the basis for these time periods is not always clearly stated [14,19,20,21,22,23]. Our 21-day observation period proved essential for accurately assessing the complete neutralizing capacity and long-term stability of novel antibody formats such as VHH.

Our results demonstrate that genetically linked trimeric VHH with different epitope specificities achieve complete neutralization, overcoming the limitations of single monoclonal antibodies. The enhancement mechanism of neutralizing activity by the trimeric structure involves three key factors. First, simultaneous binding to multiple epitopes (avidity effect) substantially strengthens binding force. TeNT has multiple functional domains including important binding sites such as the W pocket and R pocket of Fragment C, and trimeric antibodies enable efficient toxin capture through multi-site engagement. Second, simultaneous blocking of critical functional sites provides cooperative inhibition. The human monoclonal antibody TT0067 has been reported to neutralize TeNT by directly binding to the W pocket of Fragment C and inhibiting ganglioside binding [23]. VHH trimers may similarly target ganglioside binding sites and other sites necessary for cell binding and membrane translocation, cooperatively inhibiting multiple essential functions. Third, binding of multiple VHH to different sites may stabilize the overall toxin conformation, inhibiting structural changes necessary for cell invasion and cytoplasmic release of the light chain.

The inherent thermostability of VHH reduces cold chain requirements, particularly valuable for developing countries and remote veterinary practice where refrigeration infrastructure is limited or unreliable. The potential or lyophilization and room-temperature storage would further enhance accessibility in resource-limited settings and emergency response scenarios. Additionally, the small size of VHH may facilitate tissue penetration and access to toxin already bound to neuronal receptors, potentially offering therapeutic advantages beyond prophylaxis.

Susceptibility to tetanus toxin varies among animal species. Horses, cattle, sheep, goats, dogs, and others are highly susceptible, and this tVHH is particularly useful for horses, which are extremely susceptible to tetanus [1,2]. Future work should include pharmacokinetic and safety studies in target animal species, as well as strategies to extend VHH half-life such as PEGylation or Fc-fusion. This platform is applicable to other bacterial toxins such as botulinum neurotoxin and diphtheria toxin, supporting One Health initiatives bridging veterinary and human medicine.

Several limitations should be acknowledged. First, the relatively small molecular weight of VHH trimers (~45 kDa) may result in rapid renal clearance, potentially re-quiring strategies to extend serum half-life such as PEGylation, albumin binding, or Fc-fusion for therapeutic applications. Second, while our mouse model demonstrates complete protection against TeNT, pharmacokinetic and efficacy studies in target veterinary species (horses, cattle, sheep) are essential to establish optimal dosing regimens and confirm therapeutic efficacy in natural infection scenarios. Third, although TeNT structure is highly conserved across species, potential species-specific differences in antibody distribution, metabolism, and immunogenicity warrant investigation. Finally, while microbial expression offers significant advantages, large-scale GMP manufacturing processes and quality control procedures require optimization and validation for clinical implementation.

## 5. Conclusions

We developed genetically linked trivalent VHH antibodies that achieve exceptionally high neutralizing activity (approximately 1580 IU/mg) against tetanus neurotoxin through simultaneous targeting of multiple epitopes. Using an extended 21-day mouse neutralization assay, we demonstrated complete and sustained protection that overcomes the limitations of single monoclonal antibodies and physically mixed antibody combinations. This multivalent nanobody platform offers an innovative alternative to conventional immunoglobulin preparations for tetanus treatment in both veterinary and human medicine, with advantages including animal-free microbial production, enhanced biosafety, and thermostability. Alpaca VHH technology represents a next-generation antitoxin platform supporting the One Health framework, bridging veterinary and human medicine through sustainable and ethical manufacturing.

## Figures and Tables

**Table 1:** Symptom score system.

Symptom	Score
Death	0
Left/both hind limbs completely rigid, unable to walk or exhibiting severe weakness with circling movements, or abdominal rigidity and severe weakness	1
Complete paralysis of the left hind limb, with abnormal gait but active movement, or abdominal rigidity with abnormal gait	2
Marked curvature to the right side, no gait abnormality, or abdominal rigidity	3
Right scoliosis, no gait abnormality, or mild abdominal rigidity	4
Suspected right scoliosis or suspected mild abdominal rigidity	4.5
Asymptomatic	5

**Table 2:** Binding and neutralization characteristics of VHH monomers selected for trimer construction.

VHH Clone	Target Domain ᵃ	K_D to TeNT ᵇ	ELISA Binding Analysis				Estimated Neutralization Activity (IU/mg) ^c^
			TeNT	Fragment C	Fragment BC	TeTd ᵈ	
VHH-8	Fragment C	<10^−11^ M	++++	+++	++	+	≤0.0006
VHH-11	Fragment C	<10^−11^ M	++++	++++	++	++++	0.0025
VHH-36	Conformational Epitope ^e^	<10^−11^ M	++++	+	++	+++	0.005

ᵃ Determined by SPR and ELISA; ᵇ Measured by SPR; ᶜ Estimated from L+/1000 assay (40 LD_50_/mouse); ᵈ Formalin-inactivated TeNT; ^e^ Conformational epitope located at the Fragment B and Fragment C; Binding strength: ++++ Very strong; +++ Strong; ++ Moderate; + Weak.

## Data Availability

The data presented in this study are available on request from the corresponding author due to institutional data sharing agreements and ongoing collaborative research protocols.

## Supplementary Materials

The following supporting information can be downloaded at: https://www.mdpi.com/article/10.3390/vetsci13010098/s1, Figure S1: GST-VHH Fusion Protein Structure; Figure S2. Original SDS-PAGE Image for Figure 1; Figure S3. Original SDS-PAGE Image for Figure 7.

## Abbreviations

The following abbreviations are used in this manuscript:

CDR	Complementarity Determining Region
ELISA	Enzyme-Linked ImmunoSorbent Assay
EP	European Pharmacopoeia
GMP	Good Manufacturing Practice
GST	Glutathione S-transferase
IU	International Unit
KD	Dissociation Equilibrium Constant
L+	Limes Tod (limit of death dose)
LD_50_	Median Lethal Dose
PBMC	Peripheral blood mononuclear cell
SPR	Surface plasmon resonance
TeNT	Tetanus neurotoxin
TeTd	Tetanus toxoid
TIG	Human Tetanus immunoglobulin
tVHH	Trivalent variable domain of heavy chain of heavy-chain antibody
VHH	Variable domain of heavy chain of heavy-chain antibody
