# Supplementary material for: Complete Neutralization of Tetanus Neurotoxin by Alpaca-Derived Trivalent Nanobodies Aimed at Veterinary Medical Applications
Source: Vet Sci. 2026 Jan 19;13(1):98. doi: 10.3390/vetsci13010098 (PMC12846415; doi:10.3390/vetsci13010098)
Supplement: Supplementary file 1 [file vetsci-13-00098-s001.zip › vetsci-3984423-supplementary.pdf]

```
MSPILGYWKI KGLVQPTRLL LEYLEEKYEE HLYERDEGDK WRNKKFELGL
EFPNLPYYID GDVKLTQSMA IIRYIADKHN MLGGCPKERA EISMLEGAVL
DIRYGVSRIA YSKDFETLKV DFLSKLPEML KMFEDRLCHK TYLNGDHVTH
PDFMLYDALD VVLYMDPMCL DAFPKLVCFK KRIEAIPQID KYLKSSKYIA
WPLQGWQATF GGGDHPPKSD LIEGRGILQVQL...
```

Figure S1. GST-VHH Fusion Protein Structure

Schematic representation of the GST-VHH fusion protein sequence used for surface plasmon resonance analysis. The glutathione S-transferase (GST) domain is shown in gray, the Factor Xa protease cleavage site (IEGR) in red, vector-derived residues introduced during cloning (GIL) in black, and the VHH domain in blue. The VHH sequence begins with the conserved QVQL motif and continues for approximately 120 amino acids. The Factor Xa cleavage site allows for removal of the GST tag if required, leaving the linker sequences attached to the N-terminus of VHH. This fusion protein construct enables capture of VHH on anti-GST antibody-immobilized sensor chips for binding affinity measurements.

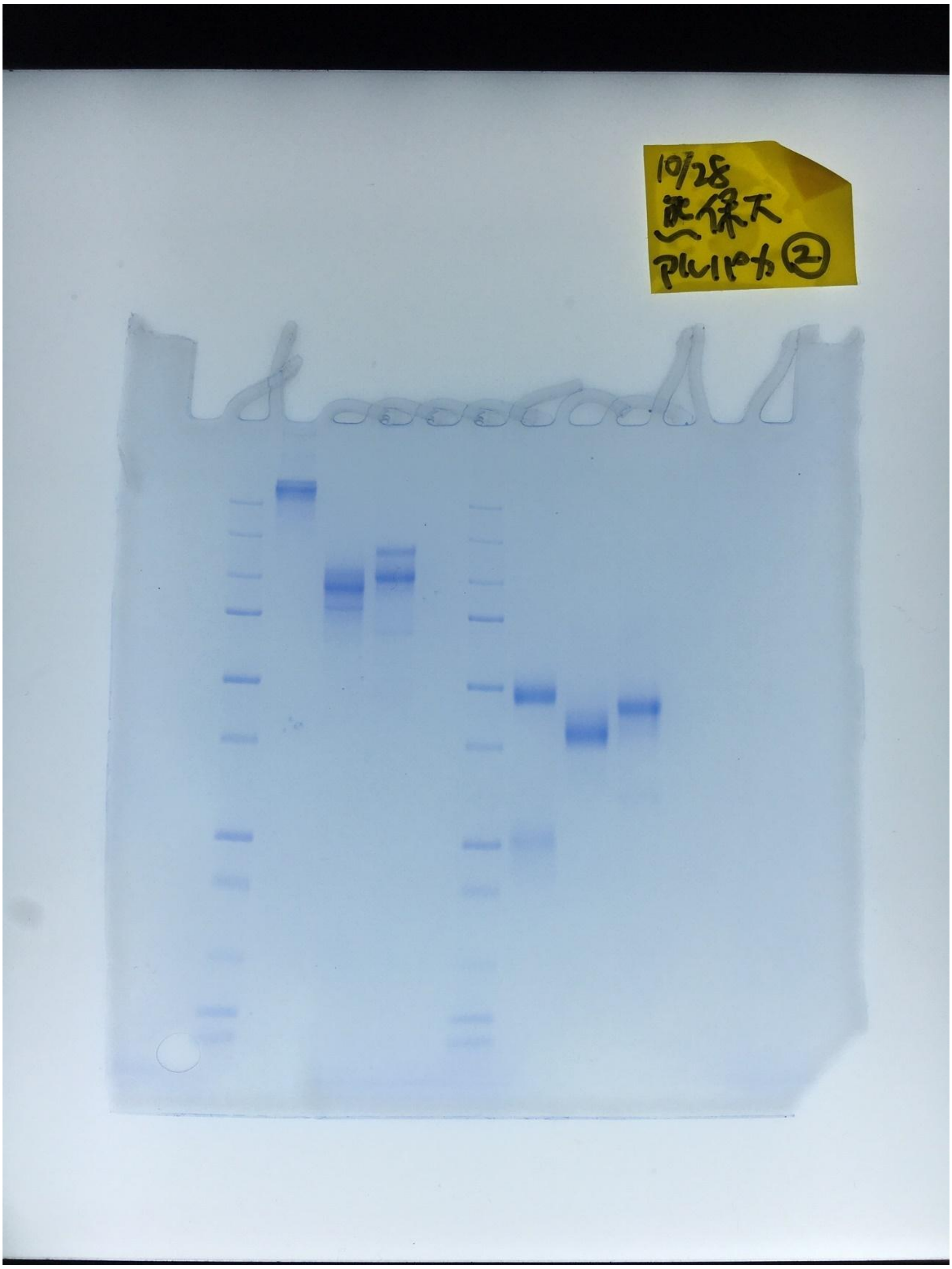

Figure S2. Original SDS-PAGE Image for Figure 1

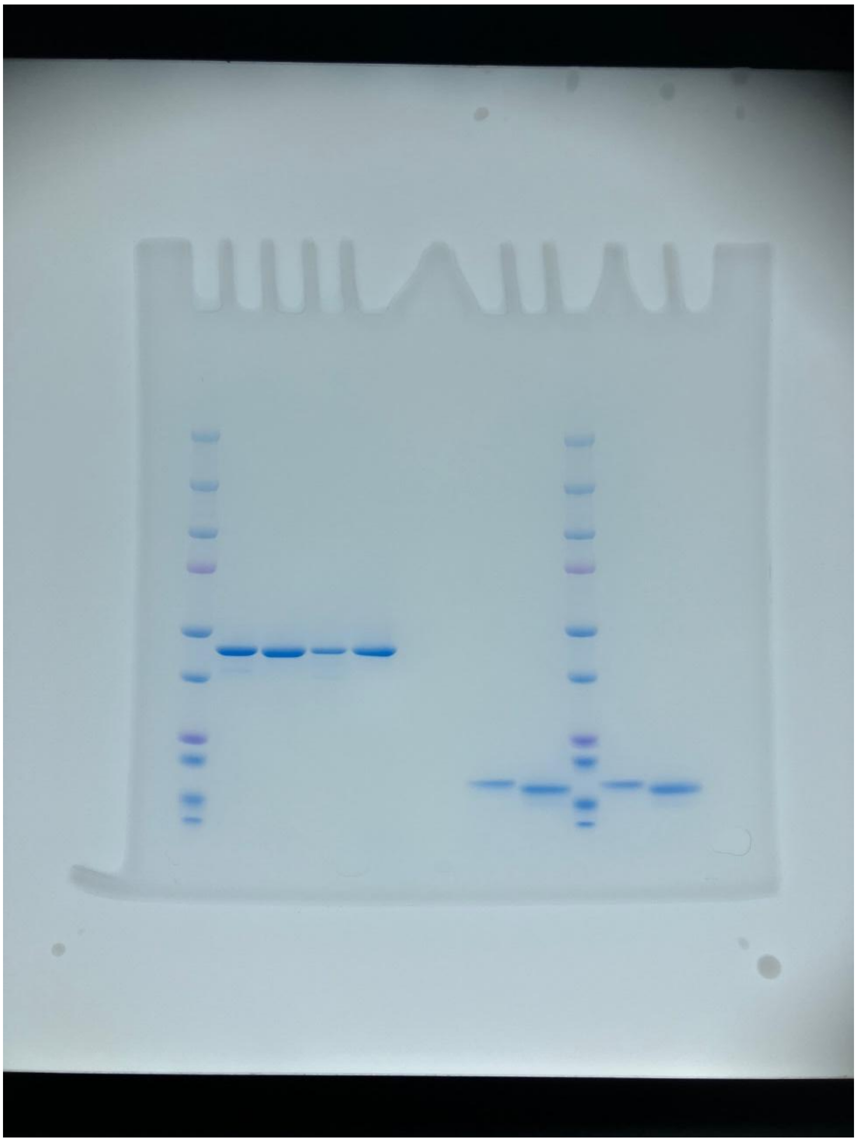

Figure S3. Original SDS-PAGE Image for Figure 7
